# Counterfinality: On the Increased Perceived Instrumentality of Means to a Goal

**DOI:** 10.3389/fpsyg.2018.01052

**Published:** 2018-07-04

**Authors:** Birga M. Schumpe, Jocelyn J. Bélanger, Michelle Dugas, Hans-Peter Erb, Arie W. Kruglanski

**Affiliations:** ^1^Social Psychology, New York University, Abu Dhabi, United Arab Emirates; ^2^Center for Health Information and Decision Systems, University of Maryland, College Park, College Park, MD, United States; ^3^Social Psychology, Helmut Schmidt University, Hamburg, Germany; ^4^Social Psychology, University of Maryland, College Park, College Park, MD, United States

**Keywords:** counterfinality, means, goals, instrumentality, goal-systems theory

## Abstract

The present research investigates the counterfinality effect, whereby the more a means is perceived as detrimental to an alternative goal, the more it is perceived as instrumental to its focal goal. The results from five studies supported this hypothesis. Study 1 demonstrated the counterfinality effect in an applied context: The more pain people experienced when getting tattooed, the more they perceived getting tattooed as instrumental to attaining their idiosyncratic goals (being unique, showing off, etc.). Study 2 experimentally replicated and extended the results of Study 1: A counterfinal (vs. non-counterfinal) consumer product was perceived as more detrimental, which in turn predicted the perceived effectiveness of the product. In Studies 3 and 5, we showed that increased perceived instrumentality due to counterfinality led to more positive attitudes toward a means. Finally, Studies 4 and 5 indicated that simultaneous commitment to both the focal and the alternative goal moderated the counterfinality effect. We discuss how various psychological phenomena can be subsumed under the general framework of counterfinality, which has broad practical implications extending to consumer behavior, health psychology, and terrorism.

## Introduction

Have you ever experienced how delicious chocolate tastes when you are on a diet or how alluring partying with friends seems while you study for an exam? Why is it that we become suddenly attracted to products or activities that undermine some of our goals? We propose that part of the answer lies in the principle of *counterfinality*, whereby the more a means (e.g., chocolate) is detrimental to an important alternative goal (e.g., dieting), the more it is perceived as instrumental to its focal goal (e.g., food enjoyment). As a result of this psychological mechanism, people grow fond of activities or products that are inherently antagonistic to alternative goals. In what follows, we examine how the principles of counterfinality operate, test the boundary conditions of counterfinality, and demonstrate how it subsumes a large number of psychological phenomena under a single framework.

## Means-End Relations: Goal-Systems Theory

The present research is grounded in Goal-Systems theory (Kruglanski et al., 2002, 2013b), which conceives of goals as knowledge structures (Hull, 1931; Tolman, 1932; Bargh, 1990; Kruglanski, 1996a). Accordingly, goals are organized into associative networks (Anderson, 1983; Anderson et al., 2004) that connect the goals to their respective means of attainment. Consistent with this proposition, the theory posits that motivational processes follow the same general principles that govern other cognitive processes.

Goal-systems theory describes several means-goal configurations. The most basic of these configurations is referred to as *unifinal*. Unifinal means are instrumental to only one goal and are unrelated to alternative goals. *Multifinal* means, on the other hand, serve multiple goals simultaneously. For example, mouthwash could be instrumental to the goal of preventing cavities and to the goal of whitening one’s teeth. One intriguing observation related to multifinal means is that because they are connected to multiple goals, they are generally perceived as less instrumental than unifinal means, a finding referred to as the dilution effect (Zhang et al., 2007).

The dilution effect is based on connectionist models, which posit that the greater the number of connections to a node, the weaker each single connection (“*fan effect*” Anderson, 1983; Anderson and Reder, 1999). Accordingly, the strength of association of a means with any *single* goal is weakened when linked to more than one goal. Given that the strength of the means-goal association is positively associated with individuals’ evaluation of the instrumentality of a means (Kruglanski, 1996b; Zhang et al., 2007), multifinal means are typically perceived as less instrumental than their unifinal counterparts. In terms of our previous example, mouthwash used to the double ends of preventing cavities and whitening one’s teeth would be perceived as less instrumental to the singular goal of whitening one’s teeth than a unifinal mouthwash that uniquely serves the goal of whitening one’s teeth. Moreover, the dilution effect is not specific to cases where additional *goals* are introduced: adding *means* to a goal results in similar outcomes (Bélanger et al., 2015).

The findings on the dilution effect support the notion that the associative strength between a means and its goal(s) follows a constant sum principle (Zhang et al., 2007). Accordingly, the more goals a means is instrumental to, the weaker are the single connections, which results in lower perceived instrumentality of the means to each individual goal. In line with this notion, Zhang et al. (2007) experimentally demonstrated that increasing the associative strength between a means and an additional goal decreased the perceived instrumentality between the means and the original goal.

Based on goal-systems theory and the logic outlined above, we propose that if a means is simultaneously instrumental to a focal goal and detrimental to another goal, it will be perceived as particularly instrumental to the focal goal it serves. In the same way that adding a positive goal connection reduces the perceived instrumentality of a means to a focal goal (i.e., dilution), adding a negative goal connection is expected to increase it (Klein, 2013; Kruglanski et al., 2015). Given that the perceived instrumentality of a means is bound by a constant sum principle, adding a negative linkage to a goal allows for a stronger link between the means and its respective focal goal (see also Kruglanski et al., 2015).

Partial evidence for this proposition comes from the work of Zhang et al. (2007), who found increased perceived instrumentality for means that could not serve additional goals. Specifically, in one of their studies, participants were asked to list things they could accomplish in the library. One group of participants was also asked to list two goals they could not accomplish in the library. The results indicated that the latter group perceived going to the library as more instrumental than the other group. Zhang et al. (2007) reasoned that the additional non-attainable goals that participants generated highlighted the uniqueness of the means-goal connections via a contrast mechanism. From our perspective, enhancing how a given means hinders the pursuit of alternative goals is a case of *counterfinality*, and the extent to which the means is detrimental to these other pursuits should play a role in how unique the means-goal association is perceived to be. Resultantly, the greater the detrimentality of the means to other goals, the greater the perceived instrumentality of this means should be. Taken together, we predict that counterfinal means will be perceived as more instrumental than unifinal means that serve the same focal goal.

## Counterfinality: A General Framework

The principle of counterfinality is a general framework under which various psychological phenomena, including psychological reactance, two-sided communication, effort justification, cognitive dissonance, and the cost heuristic, can be integrated. We discuss them in turn.

### Psychological Reactance

When products containing phosphates were forbidden in Dade County (Florida) in 1972, people started to smuggle phosphate detergent from neighboring counties or purchased the product in vast quantities (Mazis, 1973). Mazis (1975) asked the affected residents in Miami and residents in a control city (Tampa) to rate the effectiveness of phosphate laundry detergent. Miami residents rated phosphate detergents more effective regarding getting clothes clean than did residents in Tampa. Mazis (1975) explained his findings in terms of psychological reactance. *Psychological reactance* (Brehm, 1966) occurs when prohibited or unavailable products are deemed more attractive than products that can be easily obtained (see also Worchel et al., 1975). Since the use of phosphate detergent was forbidden for Miami residents, the detergent can be considered a counterfinal means. Therefore, Mazis’ (1975) findings could be explained by the principle of counterfinality.

### Two-Sided Communication

Another line of research pertinent to counterfinality is two-sided communication, that is, persuasive communication that states not only the positive but also the negative attributes of an attitudinal object. Although the findings in the literature are mixed, several studies have shown that two-sided messages lead to more favorable attitudes than messages that convey only positive information about an attitudinal object (e.g., Etgar and Goodwin, 1982; Pechmann, 1992; Bohner et al., 2003). Thus, the inclusion of negative product attributes can lead to more favorable attitudes toward an object. There are several mechanisms proposed to explain the effect, including an increased perceived credibility of the communicator due to the mentioning of negative aspects of the attitude object, as well as the *relatedness* of the positive and negative aspects (Pechmann, 1992; Bohner et al., 2003). The latter phenomenon occurs when the negative characteristics of a product reinforce its positive features because they are correlated. For instance, ice cream with a high calorie content could appear tastier than ice cream with a low calorie content (Pechmann, 1992). From a goal-systems perspective, the advertised product represents a means that is unifinal in the case of one-sided information (positive information only) and counterfinal in the case of two-sided information (negative information included). Accordingly, the counterfinality principle could explain the positive effect of two-sided communications on attitudes by the increased perceived instrumentality of the product. However, our theorizing would suggest that the counterfinality effect would still occur even if the positive and negative aspects associated with the means were not directly related to each other. Therefore, a stringent test of the counterfinality framework would entail testing whether the inclusion of negative aspects (the alternative goal to which the means is detrimental) unrelated to the positive aspects (goals to which the means is instrumental) of a means still increases the perceived instrumentality of that means (a point which we address in Study 5).

### Effort-Related Phenomena

#### Instrumentality Heuristic

Other lines of research have investigated the role of effort in shaping an individual’s evaluation of the instrumentality of a means. For example, Labroo and Kim (2009) found effortful means to be perceived as more instrumental, and hence better liked in the context of goal pursuit. Labroo and Kim (2009) argued that people tend to invest effort in means they consider instrumental to attaining their goals. As a result, during goal pursuit people may be operating under an “instrumentality heuristic” that prompts them to attribute greater instrumentality to effortful (vs. non-effortful) means. From a goal-systems perspective, effort is one of many ways through which counterfinality can be created, provided people generally hold the goal of conserving energy (see Kruglanski et al., 2012). Thus, the findings of Labroo and Kim (2009) also support our theorizing on counterfinality.

#### Inconvenience

Spiritual rituals with a greater number of procedural steps and repetitions, as well as rituals with specific time requirements, are generally perceived as more effective than rituals with fewer steps, repetitions, and undetermined execution times (Legare and Souza, 2012). From goal-systems perspective, rituals are means to specific goals, and the greater the numbers of steps and stringent requirements, the greater their detriment to the goal of convenience. In these terms, the counterfinality framework offers a theoretical explanation for why inconvenient rituals are perceived as more instrumental than convenient ones.

#### Cognitive Dissonance

The aforementioned findings bear similarities to the concept of *effort justification* (Festinger, 1957), which is the tendency to attribute greater value to an outcome if one had to invest a great deal of effort into attaining it. Festinger (1957) *cognitive dissonance theory* states that whenever individuals engage in an unpleasant activity to obtain a specific outcome, they experience an aversive state of cognitive dissonance because the cognition that the activity is unpleasant is considered dissonant with engaging in the activity. In order to reduce cognitive dissonance, the outcome is perceived as more desirable, which adds consonant cognitions (Festinger, 1957). For instance, university students who experienced a humiliating initiation to join a group expressed more liking for the group than students who did not go through that experience (Aronson and Mills, 1959). From the perspective of cognitive dissonance theory, the cognition that the initiation was humiliating is dissonant with joining the group. Therefore, consonant cognitions are added, and the group is perceived as more desirable. From our current perspective, joining the group is detrimental to the goal of avoiding humiliation, and the favorable evaluation of the group can be explained by increased perceived instrumentality due to the counterfinal goal-means configuration. Thus, phenomena previously explained by cognitive dissonance theory (Festinger, 1957) can be explained by and subsumed under the counterfinality framework.

Importantly, the theory of counterfinality provides a broader framework than cognitive dissonance theory, as it captures a broader range of cases, such as those discussed previously. Furthermore, dissonance is (by definition) limited to discrepancies between attitudes and behaviors (Festinger, 1957); however, counterfinality also captures “mental conflicts,” that is, situations in which individuals encounter goal-conflicting information. For example, learning that an activity one desires to engage in will be effortful or that a product one intends to use will have some painful side effects can result in increased perceived instrumentality, given that one subscribes to the goal of convenience or avoiding pain. Thus, for the counterfinality effect to occur, it is not a necessary condition that individuals engage in a certain behavior. Moreover, counterfinality can also explain *why* people engage in detrimental behaviors (because they are perceived as instrumental), whereas cognitive dissonance theory explains why people adjust their cognitions *after* they have engaged in detrimental behaviors.

### Other Phenomena

A plurality of other phenomena can also be explained through the principles of counterfinality. For example, the counterfinality effect could help explain the underlying appeal of temptations (Fishbach et al., 2003), e.g., highly caloric foods contrary to one’s diet, substances detrimental to one’s health, or risky behaviors such as extreme sports of various kinds potentially resulting in injury or death. Relatedly, it was found that presenting food as healthy to children decreased their food enjoyment (Maimaran and Fishbach, 2014). Another phenomenon relevant to counterfinality is the use of cost-related information to make inferences about the quality or efficacy of an item or behavior (termed *cost heuristic*, Klein, 2013; Klein, 2011, Unpublished). Generally, items with a higher (vs. lower) price tag are perceived to be of higher quality (Gerstner, 1985; Rao and Monroe, 1989). From a goal-systems perspective, a product or means would be perceived as more instrumental (and hence better liked) due to its counterfinal relation to the goal of saving money. An interesting prediction derived from the counterfinality framework is that the more important the alternative goal, the stronger the counterfinality effect. Thus, an expensive product should be perceived as instrumental, and even more so, when the goal of saving money is deemed important. Likewise, the relationship between effortful (vs. effortless) means and instrumentality should be even stronger when the goal of convenience is important. A detrimental effect on an important alternative goal would imply greater counterfinality and hence lead to higher perceived instrumentality of the means. Last, but not least, focal goal magnitude is also hypothesized to moderate the counterfinality effect: Without attributing substantial importance to the focal goal, it is unlikely that people would be willing to sacrifice alternative concerns, especially in important life domains (e.g., Bélanger et al., 2014).

Overall, a wide range of situations can influence individuals’ evaluation of the instrumentality of a means. As can be gleaned from the present literature review, several separate explanations of these phenomena have been proposed (e.g., psychological reactance, relatedness of positive and negative aspects in two-sided communications, invested effort, and monetary cost). However, these different lines of research share a common denominator: They all involve a means instrumental to a focal goal that is also detrimental to an alternative goal. Thus, our model of counterfinality “connects the dots” in that it subsumes these mechanisms under a unifying framework, whereby the more a means is detrimental to an alternative goal, the more it is perceived as instrumental to the focal goal. Furthermore, compared to other frameworks, our theoretical approach specifies the conditions under which this effect should be more pronounced, that is, when individuals are concomitantly committed to the focal and the alternative goal. Finally, in line with Labroo and Kim (2009), we expected that the increased instrumentality of a means due to counterfinality would lead to greater liking of the means.

## The Present Research

The foregoing predictions were tested in five studies conducted in a variety of contexts to demonstrate the generalizability of the counterfinality effect. In Study 1, we tested the hypothesis that the more a means is detrimental to an alternative goal, the more it is perceived as instrumental to the focal goal. The aim of Study 2 was to replicate Study 1 using an experimental design comparing counterfinal to unifinal means and to test whether the extent to which the means is detrimental to the alternative goal mediates the relationship between the type of the means (counterfinal vs. unifinal) and its perceived instrumentality. Relatedly, Studies 3 and 5 tested whether perceived instrumentality translates into greater liking of the means. Studies 4 and 5 aimed to show that the counterfinality effect is most pronounced when the means is highly detrimental to the alternative goal and when individuals attribute high importance to both the focal and alternative goals.

## Study 1

The purpose of Study 1 was to document the counterfinality effect in the context of tattooing. People get tattooed for a wide range of reasons and we asked people why they got tattooed (focal goal). We hypothesized that the more people experienced pain while getting tattooed, the more effective they would perceive tattooing to attaining their idiosyncratic focal goal.

### Materials and Methods

#### Participants and Design

A total of 96 participants from the United States (41 women, 55 men; *M*_age_ = 31.57, *SD*_age_ = 10.96) took part in this study, ostensibly to study peoples’ motivations for getting tattooed. The study explicitly asked for participants who had at least one tattoo.

#### Procedure and Materials

Participants were recruited on Amazon’s Mechanical Turk (Mturk) and provided with a link to the survey. We asked participants for their main motivation for getting tattooed and provided a box for them to type in their answer. Then, participants indicated the extent to which they agreed with the following statement “Getting tattooed was a bit painful” (1 = *Strongly disagree*, 10 = *Strongly agree*). Participants were also asked, “How effective was getting tattooed for achieving your main goal of [BLANK]?” (we inserted the goal participants listed earlier; 1 = *Not effective at all*, 10 = *Very effective*). Finally, we asked participants to indicate their age and gender. Upon completion, participants were debriefed and paid through MTurk.

### Results and Discussion

Participants indicated various reasons for getting tattooed, such as “to rebel against my parents,” “to connect the tattoo to a specific memory,” or “to show off.” Participants’ reported pain for getting tattooed was entered into a regression model to predict the perceived effectiveness of getting tattooed for the idiosyncratic goal they listed. The overall model was significant, *F*(1,94) = 19.92, *p* < 0.001, *R*^2^ = 0.18. Pain significantly predicted how effective getting tattooed was perceived, β = 0.42, *t*(94) = 4.46, *p* < 0.001. We display means, standard deviations, and correlations for all measures in **Table 1**.

**Table 1:** Means, standard deviations, and correlations involving all variables from Study 1 (*N* = 96).

	*M*	*SD*	2
Pain (1)	6.35	2.53	0.42^∗^
Effectiveness (2)	8.08	2.14	

*^∗^p < 0.001.*

Thus, Study 1 provides initial support for the counterfinality effect, namely that the more painful a means was experienced, the more it is perceived as instrumental to people’s idiosyncratic focal goal. In Study 1, this relationship was demonstrated in an applied setting, with participants having actually experienced an instance of counterfinality. Next, we designed an experiment to test the mechanism behind the counterfinality effect in a laboratory setting.

## Study 2

The objectives of Study 2 were twofold: (1) to provide evidence for the *causal* influence of counterfinality on means’ perceived instrumentality and (2) to highlight the psychological mechanism at play. To that end, we conducted an experiment to test the hypothesis that counterfinal means are perceived as more instrumental than unifinal ones. We expected that the extent to which the means is perceived as detrimental would mediate this effect.

### Materials and Methods

#### Participants and Design

Forty-nine undergraduate students (32 women, 17 men; *M*_age_ = 19.14, *SD*_age_ = 1.89) at a large mid-Atlantic university in the United States took part in a study on “evaluation of consumer products” to fulfill course requirements^1^. They were randomly assigned to one of two experimental conditions (unifinal vs. counterfinal) in a between-subjects design.

#### Procedure and Materials

Participants were asked to read about a mouthwash that supposedly reduced the likelihood of getting a sore throat. Participants were presented with the product label of “Oral Defense Rinse” that described the mouthwash as paraben and alcohol free as well as offering a cool mint taste^2^. In the unifinal (control) condition, the label had the slogan “No burn - still kills the germs.” In contrast, participants in the counterfinal condition were presented with a slightly different slogan “It burns – but it kills the germs.” Participants were asked to evaluate the mouthwash and then answered some demographic questions (age, gender). Lastly, they were debriefed and dismissed.

##### Manipulation check

To probe whether the mouthwash in the counterfinal condition was indeed perceived as creating a burning sensation, we asked participants to indicate their level of agreement with the statement “Using the mouthwash will probably create a burning sensation in my mouth” (−10 = *Strongly disagree*, 10 = *Strongly agree*).

##### Instrumentality measure

We used the following two items (α = 0.94) to assess the perceived instrumentality of the mouthwash for its claimed main purpose of fighting germs that cause sore throats: “I believe that the mouthwash kills germs that cause sore throats,” as well as “I believe that the mouthwash helps prevent sore throats” (−10 = *Strongly disagree*, 10 = *Strongly agree*).

### Results and Discussion

**FIGURE 1:**
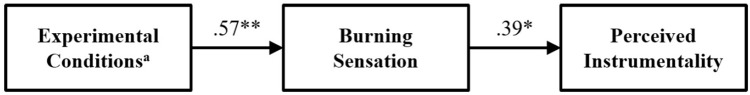
Results from the path analysis (Study 2). ^a^0 = unifinal means; 1 = counterfinal means. ^∗∗^*p* < 0.001, ^∗^*p* < 0.05.

Path analyses were conducted to investigate the mediating role of burning sensation between the type of means-goal configurations and instrumentality. The model was tested with AMOS (Arbuckle, 2007) using maximum likelihood estimation procedure. Two paths were specified: One path from the experimental condition (coded 0 for the unifinal means and 1 for the counterfinal means) to perceptions of burning sensation, and one path linking burning sensation to perceived instrumentality (see **Figure 1**). We display means, standard deviations, and correlations for all measures in **Table 2**. Results revealed that the hypothesized model fit the data well, χ^2^(*df* = 1, *N* = 49) = 2.17, *p* = 0.14, GFI = 0.97, CFI = 0.96, IFI = 0.96, RMSEA = 0.16, AIC = 12.17.

**Table 2:** Means, standard deviations, and correlations involving all variables from Study 2 (*N* = 49).

	*M*	*SD*	2	3
Condition^a^ (1)	0.51	0.51	0.57^∗^	0.38^∗^
Burning sensation (2)	1.73	6.61		0.39^∗^
Instrumentality (3)	3.86	4.45		

*^∗^p < 0.05. ^a^0 = unifinal means; 1 = counterfinal means.*

All estimated paths were significant. The experimental condition was positively related to burning sensation (β = 0.57, *p* < 0.001), and burning sensation was positively associated to instrumentality (β = 0.39, *p* = 0.004). Bootstrapped confidence interval estimates of the indirect effect (see Preacher and Hayes, 2008) were calculated to confirm the significance of mediation. The 95% confidence interval of the indirect effect was obtained with 5000 bootstrap resamples (Preacher and Hayes, 2008). Results confirmed the mediating role of burning sensation between type of means-goal configurations and perceived instrumentality (β = 0.22; CI = 0.038–0.421).

The hypothesized model was tested against an alternative model, whereby means-goal configurations predicted perceived instrumentality, which in turn predicted perceived burning sensation. The model tested the possibility that a counterfinal product would be perceived as more instrumental than its unifinal counterpart, and therefore, perceived as causing a stronger burning sensation. Consistent with our theory, the alternative model yielded worse fit indices than the hypothesized model, χ^2^(*df* = 1, *N* = 49) = 13.66, *p* < 0.001, GFI = 0.86, CFI = 0.51, IFI = 0.55, RMSEA = 0.51, AIC = 23.66.

In summary, Study 2 provides experimental support for the counterfinality effect and demonstrates the psychological mechanism through which it occurs. Specifically, the counterfinal mouthwash was perceived as causing a burning sensation, which in turn led to heightened perceptions of instrumentality. Next, we set out to demonstrate that counterfinal means are preferred because they are perceived as more instrumental. This would support a new psychological mechanism to explain the positive effect of two-sided communications.

## Study 3

The objectives of Study 3 were to replicate Study 2 and to test whether enhancing the perceived instrumentality of a product for a goal it purportedly serves would influence the extent to which people like and want to purchase the product. If a counterfinal product would be better liked due to its increased perceived instrumentality, then the counterfinality effect could offer an explanation as to how two-sided communications–that signify counterfinality–foster more favorable attitudes than one-sided communications.

### Materials and Methods

#### Participants and Design

Two hundred United States participants (92 women, 108 men; age: *M*_age_ = 30.00, *SD*_age_ = 7.65) were recruited via MTurk to take part in a study on consumer products. They were randomly assigned to one of two experimental conditions (unifinal vs. counterfinal) in a between-subjects design.

#### Procedure and Materials

Participants read an excerpt ostensibly taken out of a scientific report. They learned that researchers found out that gas-emitting bacteria on the tongue were responsible for bad breath. Therefore, a new mouthwash that eliminates the bacteria responsible for bad breath was developed. Subsequently, participants were asked to study a product label concerning that mouthwash and to evaluate the mouthwash on several dimensions. Participants were presented with either a unifinal (control) or a counterfinal version of a mouthwash – a manipulation that had proven successful in Study 2. Then, participants were asked to indicate their age and gender. Lastly, they were debriefed, thanked, and paid for their participation.

##### Instrumentality

We asked participants to indicate their level of agreement with the following statement: “I believe that the mouthwash eliminates bacteria that are responsible for bad breath” (−10 = *Strongly disagree*, 10 = *Strongly agree*).

##### Attitude measures

We asked participants, “How much do you like the mouthwash (−10 = *Dislike*, 10 = *Like*),” and “How likely would you buy the mouthwash” (0 = *Very unlikely*, 10 = *Very likely*). We standardized the two variables and created an overall attitude measure (α = 0.91).

### Results and Discussion

**FIGURE 2:**
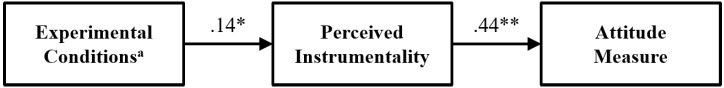
Results from the path analysis (Study 3). ^a^0 = unifinal means; 1 = counterfinal means. ^∗∗^*p* < 0.001, ^∗^*p* < 0.05.

Path analyses were conducted to examine the mediating role of perceived means instrumentality of the relation between means-goal configurations (counterfinal vs. unifinal) and attitudes. The model was tested with AMOS using maximum likelihood estimation procedure. Two paths were specified: One path from the experimental conditions (coded 0 for the unifinal means and 1 for the counterfinal means) to perceived instrumentality, and one path linking perceived instrumentality to attitudes (see **Figure 2**). We display means, standard deviations, and correlations for all measures in **Table 3**. Results revealed that the hypothesized model fits the data well, χ^2^(*df* = 1, *N* = 200) = 1.25, *p* = 0.26, GFI = 0.99, CFI = 0.99, IFI = 0.99, RMSEA = 0.04, AIC = 11.25.

**Table 3:** Means, standard deviations, and correlations involving all variables from Study 3 (*N* = 200).

	*M*	*SD*	2	3
Condition^a^ (1)	0.50	0.50	0.14^∗^	−0.01
Instrumentality (2)	5.38	4.09		0.44^∗^
Attitudes (3)	0.00	0.96		

*^∗^p < 0.05. ^a^0 = unifinal means; 1 = counterfinal means.*

The experimental condition variable was positively related to perceived instrumentality (β = 0.14, *p* = 0.04), which in turn was positively associated with attitudes (β = 0.44, *p* < 0.001). As in Study 2, bootstrapped confidence interval estimates of the indirect effect (see Preacher and Hayes, 2008) were calculated to confirm the significance of mediation. Results confirmed the mediating role of perceived instrumentality between the type of means-goal configuration (counterfinal vs. unifinal) and attitudes (β = 0.06; CI = 0.003–0.133).

The hypothesized model was tested against an alternative model, whereby means-goal configurations predicted attitudes, which in turn predicted perceived instrumentality. The model tested the possibility that a counterfinal product would be better liked than its unifinal counterpart, and therefore, perceived as more instrumental. Consistent with our theory, the alternative model had worse fit indices than the hypothesized model, χ^2^(*df* = 1, *N* = 200) = 5.42, *p* = 0.02, GFI = 0.98, CFI = 0.90, IFI = 0.91, RMSEA = 0.15, AIC = 15.42.

The results of Study 3 support the notion that counterfinal (vs. unifinal) products are positively evaluated in terms of liking and purchase intentions because they are perceived as more instrumental to the goal they serve. Thus, these findings demonstrate that the counterfinality effect offers a viable possible explanation for the positive effect of two-sided communications on attitudes.

One limitation of our empirical findings so far, is that we have assumed that individuals generally want to avoid pain, and hence, perceive its experience as counterfinal. Another assumption is that participants had been at least moderately committed to the focal goal relevant to each experiment. However, individual differences on these dimensions should appropriately affect the magnitude of the counterfinality effect. Study 4 aimed to investigate this implication of our reasoning.

## Study 4

The objective of Study 4 was to examine the moderating role of alternative and focal goal magnitude. We expected the counterfinality effect to be more pronounced when both the alternative as well as the focal goal are of high importance for the individual, as outlined in the theoretical part of this research.

### Materials and Methods

#### Participants and Design

We recruited 144 participants (81 women, 62 men, one person preferred not to disclose gender; *M*_age_ = 33.58, *SD*_age_ = 10.01) from the United States via MTurk to take part in a study on fitness. The alleged purpose of the study was to help with a marketing strategy promoting a fitness program.

#### Procedure and Materials

Participants were asked to evaluate a fitness program for burning fat based on testimonials provided by other people (filler statement in both conditions: “In the course of this 3-week program, I lost 10 lbs”). In the counterfinal condition (“It was a good workout, 3 times a week. Each session lasted for 30 min and I definitely felt it everywhere,” “The pain in my body afterward was excruciating”), the program was described as more painful than in the unifinal condition (“It was a good workout, 3 times a week. Each session lasted for 30 min,” “I felt it in my body afterward”).

##### Manipulation check

To test whether the fitness program in the counterfinal condition was indeed perceived as more painful, we asked participants to indicate their level of agreement with the following two statements (α = 0.92): “Taking part in this fitness program probably involves a lot of pain,” as well as “I believe this fitness program goes hand in hand with a lot of pain” (0 = *Strongly disagree*, 10 = *Strongly agree*).

##### Instrumentality measure

The following question measured participants’ perceived instrumentality of the fitness program, “How effective do you think this training program is with regards to burning fat” (0 = *Very ineffective*, 10 = *Very effective*).

##### Focal goal magnitude

The importance of the focal goal was measured with the following items: “How often do you work out?” (1 = *Never*, 5 = *More than five times a week*), “Getting a good workout regularly is important to me” (0 = *Strongly disagree*, 10 = *Strongly agree*) and “How important is it for you to be in shape?” (0 = *Not at all important*, 10 = *Extremely important*). Items were standardized and combined into a focal goal magnitude index (α = 0.84).

##### Alternative goal magnitude

Two items (α = 0.66) measured the importance of the alternative goal: “How important is it for you to not feel any pain after working out?” and “In general, how important is the goal of comfort for you in regards to fitness and exercising?” (0 = *Not at all important*, 10 = *Extremely important*).

### Results and Discussion

A manipulation check confirmed that the fitness programs differed in terms of perceived painfulness. Results indicated that the counterfinal fitness program (*M* = 6.26, *SD* = 2.14) was perceived as more painful than the unifinal one (*M* = 4.44, *SD* = 2.33), *F*(1,142) = 23.87, *p* < 0.001, η^2^ = 0.14. We display means, standard deviations, and correlations for all measures in **Table 4**. Hierarchical multiple regression analyses were conducted to examine the simple as well as interaction effects between perceived painfulness of the fitness program (manipulation check measure), focal goal magnitude (importance of fitness), and alternative goal magnitude (importance of comfort/avoidance of pain). According to Aiken and West (1991) procedures, independent variables were standardized before calculating the interaction products. We entered painfulness, alternative goal magnitude, as well as focal goal magnitude in Step 1, their corresponding two-way interactions in Step 2, and the three-way interaction in Step 3. Step 1 explained a significant amount of variance in instrumentality, *F*(3,140) = 6.33, *p* < 0.001, *R*^2^ = 0.12. Results showed that painfulness (β = 0.27, *p* < 0.001) as well as focal goal magnitude (β = 0.29, *p* = 0.005) were positively related to instrumentality, whereas alternative goal magnitude had no significant effect on instrumentality (β = 0.07, *p* = 0.36). The addition of the two-way interaction terms in Step 2 increased explained variance only marginally, *F*(3,137) = 2.23, *p* = 0.09, Δ*R*^2^ = 0.04. Results showed that the interaction between Focal Goal Magnitude × Alternative Goal Magnitude (β = −0.14, *p* = 0.081) as well as the interaction between Alternative Goal Magnitude × Painfulness (β = 0.15, *p* = 0.057) almost reached significance, whereas the interaction between Focal Goal Magnitude × Painfulness did not (β = 0.06, *p* = 0.448). Most importantly, adding the three-way interaction (β = 0.22, *p* = 0.009) in Step 3 significantly increased explained variance, *F*(1,136) = 6.98, *p* = 0.009, Δ*R*^2^ = 0.04 (see **Table 5**).

**Table 4:** Means, standard deviations, and correlations involving all variables from Study 4 (*N* = 144).

	*M*	*SD*	2	3	4	5
Condition^a^ (1)	0.52	0.50	0.38^∗∗^	0.22^∗^	0.08	−0.10
Painfulness (2)	5.39	2.40		0.26^∗^	−0.09	0.13
Instrumentality (3)	6.87	2.06			0.19^∗^	0.07
FGM (4)	0.00	.87				−0.16^∗^
AGM (5)	5.06	2.08				

*^∗∗^p < 0.001; ^∗^p < 0.05. ^a^0 = unifinal means; 1 = counterfinal means; FGM, focal goal magnitude; AGM, alternative goal magnitude.*

**Table 5:** Results of regressions predicting instrumentality from pain (P), alternative goal magnitude (AGM) and focal goal magnitude (FGM) in Study 4 (*N* = 144).

	*F*	*R*^2^	Δ*R*^2^	*P*	AGM	FGM	PxAGM	PxFGM	AGMxFGM	PxAGMxFGM
Step 1	6.33^∗∗^	0.12	0.12	0.27^∗^	0.07	0.29^∗^	–	–	–	–
Step 2	2.23^∗^	0.16	0.04	0.25^∗^	0.08	0.22^∗^	0.15	0.06	−0.14	
Step 3	6.98^∗^	0.20	0.04	0.25^∗^	0.08	0.16	0.16^∗^	0.11	−0.20^∗^	0.22^∗^

*^∗^p < 0.05, ^∗∗^p < 0.001.*

**FIGURE 3:**
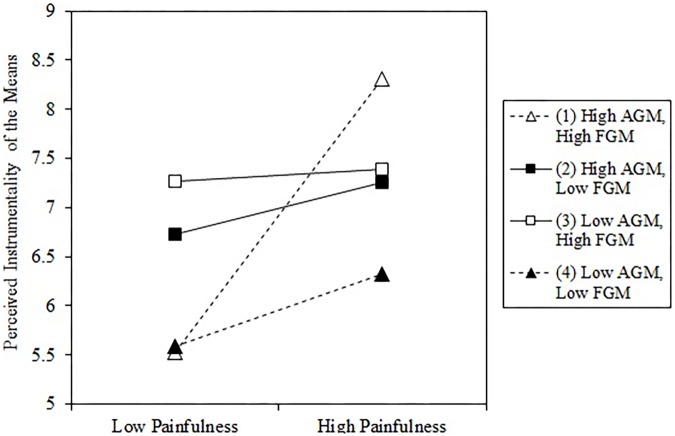
Perceived instrumentality for low vs. high painfulness of a fitness program (means) dependent on focal goal magnitude (FGM) and alternative goal magnitude (AGM) (Study 4). High = one standard-deviation higher than the mean; Low = one standard-deviation lower than the mean.

To further probe the nature of the three-way interaction, we computed the conditional effect of the Painfulness × Alternative Goal Magnitude interaction for low vs. high levels of focal goal magnitude (Hayes, 2013). The interaction Painfulness × Alternative Goal Magnitude was significant for high levels (1 *SD* above the mean) of focal goal magnitude (*b* = 0.66, 95% CI [0.26, 1.07], *t*(136) = 3.24, *p* = 0.002) but nor for low levels (1 *SD* below the mean) of focal goal magnitude (*b* = −0.05, 95% CI [−0.44,0.34], *t*(136) = −0.26, *p* = 0.797). Results of simple slope analyses (Aiken and West, 1991) revealed that the effect of painfulness on perceived instrumentality was more acute for individuals high in alternative and focal goal magnitude (see **Figure 3**). Only the slope for high alternative goal magnitude and high focal goal magnitude (*b* = 1.39, 95% CI [0.76,.2.02], *t*(136) = 4.39, *p* < 0.001) was significant (all other *p*s > 0.05). Thus, painfulness of the fitness program was associated with increased perceptions of instrumentality for individuals high in focal goal magnitude (importance of fitness) and high in alternative goal magnitude (importance of avoiding pain). Although the results of Study 4 supported our predictions with the alternative goal of avoiding pain, the conceptualization of counterfinality is much broader and should hold for various alternative goals. This is why we set out to demonstrate the breadth of the counterfinality framework in our next study.

## Study 5

In Study 5, we further investigated the moderating role of goal magnitude in another context. This time, instead of predetermining the alternative goal, participants generated the alternative goal to which the means was detrimental. The purpose of this approach was to demonstrate that counterfinality applies to any kind of goal-pursuit. Additionally, we aimed to demonstrate that our findings still hold after controlling for the relationship between the focal and the alternative goal. By doing so, we aim to address the alternative explanation that a detrimental effect on an alternative goal (e.g., muscle soreness) could have increased the perceived instrumentality of the means to achieve the focal goal (e.g., burning fat) only because the two are correlated (i.e., muscle soreness signals muscle growth, which in turn leads to burning more calories, and hence fat). The proposition that a negative and positive aspect of an attitudinal object are related and therefore reinforce each other has been put forward in the communication literature to explain the positive effect of two-sided communication (Pechmann, 1992). The counterfinality model, on the other hand, contends that greater perceived means instrumentality should occur as long as the means is detrimental to the alternative goal, even when controlling for a possible relationship between the focal and the alternative goal.

### Materials and Methods

#### Participants and Design

Two hundred and three participants from the United States (103 women, 100 men; *M*_age_ = 32.79, *SD*_age_ = 8.56) were recruited via MTurk. To be part of the study, participants needed at least one tattoo and must have perceived getting tattooed as detrimental to an alternative goal. Another sample of 48 participants (25 women, 23 men; *M*_age_ = 41.94, *SD*_age_ = 12.80) recruited on MTurk served as independent and naïve raters for the content that the 203 participants listed. We asked this latter group of participants to rate the extent to which each of the alternative and focal goals listed by the first group of participants (*N* = 203) were related.

#### Procedure and Materials

Participants were asked to indicate their most important goal for getting tattooed, how effective they thought getting tattooed was for achieving that goal, as well as how important this goal was to them. Next, we asked participants to think of and write down a goal to which getting tattooed was detrimental, to rate the extent to which they perceived getting tattooed was detrimental to that goal, as well as to rate the importance of that goal. This procedure allowed to measure the extent to which a means is counterfinal in domains generated by the participants rather than in experimentally predetermined domains.

##### Instrumentality measure

Participants were asked to indicate how effective getting tattooed was for achieving their idiosyncratic focal goal (1 = *Not effective at all*, 10 = *Very effective*).

##### Attitude measures

We assessed the number of tattoos as a behavioral attitude measure (the greater the number of tattoos, the more favorable the attitude toward tattooing).

##### Focal goal magnitude

After indicating their idiosyncratic focal goal, we asked participants how important this goal was to them (1 = *Not important at all*, 10 = *Very important*).

##### Alternative goal magnitude

We further asked participants how important to them was the alternative goal listed (1 = *Not important at all*, 10 = *Very important*).

##### Detrimentality

Participants rated the extent to which getting tattooed was detrimental to their alternative goal (1 = *Not detrimental at all*, 10 = *Very detrimental*).

##### Relatedness

The raters were asked to indicate the extent to which the means’ negative impact on the alternative goal prevented or helped focal goal pursuit (−4 = *negative impact on alternative goal prevented focal goal pursuit*, 0 = *not connected at all*, +4 = *negative impact on alternative goal helped focal goal pursuit*). The intraclass correlation coefficient (ICC) indicated high inter-judge reliability (ICC = 0.89), therefore we averaged the raters’ scores for each pair of alternative and focal goal.

Finally, we asked all participants to indicate their age and gender. Upon completion, participants were debriefed and paid through their MTurk accounts.

### Results and Discussion

Participants listed various focal goals for getting tattooed (e.g., looking attractive, self-expression, or remembering a special event or person), as well as numerous alternative goals they perceived getting tattooed was detrimental to (e.g., looking professional, getting a job, saving money).

**FIGURE 4:**
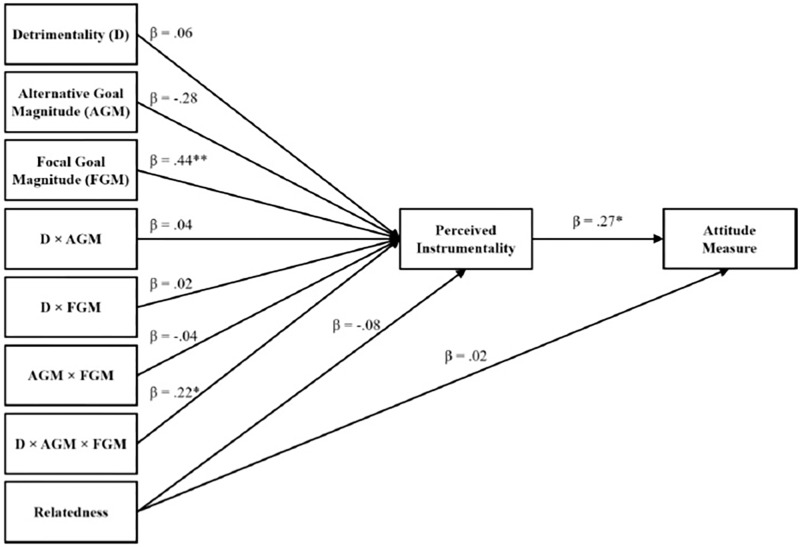
Indirect effect of Detrimentality. Alternative Goal Magnitude. Focal Goal Magnitude, as well as their interaction terms on attitudes through perceived instrumentality, controlling for Relatedness of the Focal Goal and Alternative Goal (Study 5).

Path analyses were conducted to investigate the mediating role of perceived instrumentality between the three-way interaction (Detrimentality × Alternative Goal Magnitude × Focal Goal Magnitude) and attitudes. The model was tested with AMOS (Arbuckle, 2007) using maximum likelihood estimation procedure. Ten paths were specified: The simple effects, two-way interactions, and the three-way interaction of the independent variables predicting instrumentality (seven paths). Furthermore, two paths were specified between relatedness and instrumentality and attitudes. Lastly, a path was specified from instrumentality to attitudes (see **Figure 4**). We display means, standard deviations, and correlations for all measures in **Table 6**. Results revealed that the hypothesized model fits the data well, χ^2^(*df* = 7, *N* = 203) = 5.33, *p* = 0.62, GFI = 1.00, CFI = 1.00, IFI = 1.00, RMSEA = 0.00, AIC = 101.33.

**Table 6:** Means, standard deviations, and correlations involving key variables from Study 5 (*N* = 203).

	*M*	*SD*	2	3	4	5	6
Detrimentality (1)	5.34	2.71	0.16^∗^	0.10	0.09	0.12	0.19^∗^
AGM (2)	6.11	3.28		0.08	0.02	−0.01	0.23^∗^
FGM (3)	8.21	2.24			0.45^∗∗^	0.10	−0.06
Instrumentality (4)	8.24	1.99				0.18^∗^	−0.07
Attitude (5)	2.65	3.03					0.01
Relatedness (6)	−0.57	0.70					

*^∗^p < 0.05, ^∗∗^p < 0.001. FGM, focal goal magnitude; AGM, alternative goal magnitudes.*

To analyze the a-path of this mediation model, we tested the three-way interaction (Detrimentality × Alternative Goal Magnitude × Focal Goal Magnitude) on instrumentality using hierarchical multiple regression analyses. According to Aiken and West (1991) procedures, independent variables were standardized before calculating the interaction products. To control for relatedness of the alternative and focal goals, we also included the raters’ mean scores as a control variable into the model in Step 1. We entered detrimentality, alternative goal magnitude, as well as focal goal magnitude in Step 2, their corresponding two-way interactions in Step 3, and the three-way interaction in Step 4. Step 1 did not explain a significant amount of variance in instrumentality, *F*(1,201) = 0.89, *p* = 0.346, *R*^2^ = 0.00; the relatedness of the alternative and focal goal was not predictive of perceived instrumentality (β = −0.08, *p* = 0.346). Step 2 increased explained variance significantly, *F*(3,198) = 16.88, *p* < 0.001, Δ*R*^2^ = 0.20. Detrimentality (β = 0.06, *p* = 0.374) as well as alternative goal magnitude (β = −0.28, *p* = 0.779) were not related to instrumentality, but focal goal magnitude was a positive predictor of instrumentality (β = 0.44, *p* < 0.001). The addition of the two-way interaction terms in Step 3 did not increase explained variance, *F*(3,195) = 0.26, *p* = 0.854, Δ*R*^2^ = 0.00; none of the two-way interactions was significant (Focal Goal Magnitude × Alternative Goal Magnitude, β = −0.04, *p* = 0.546; Alternative Goal Magnitude × Detrimentality, β = 0.04, *p* = 0.510; Focal Goal Magnitude × Detrimentality, β = 0.02, *p* = 0.814). Most importantly, adding the three-way interaction (β = 0.22, *p* = 0.002) in Step 4 significantly increased explained variance, *F*(1,194) = 9.69, *p* = 0.002, Δ*R*^2^ = 0.04 (see **Table 7**).

**Table 7:** Results of regressions predicting instrumentality from detrimentality (D), alternative goal magnitude (AGM) and focal goal magnitude (FGM) controlling for relatedness of the goals (Relat.) (*N* = 203).

	*F*	*R*^2^	Δ*R*^2^	Relat.	*D*	AGM	FGM	DxAGM	DxFGM	AGMxFGM	DxAGMxFGM
Step 1	0.89	0.00	0.00	−0.07	–	–	–	–	–	–	–
Step 2	16.88^∗∗^	0.21	0.20	−0.05	0.06	−0.28	0.44^∗∗^	–	–	–	–
Step 3	0.26	0.21	0.00	−0.05	0.06	−0.01	0.24^∗^	0.04	0.02	−0.04	
Step 4	9.69^∗^	0.25	0.04	−0.08	0.06	−0.03	0.43^∗∗^	−0.04	0.06	−0.03	0.22^∗^

*^∗^p < 0.05, ^∗∗^p < 0.001.*

**FIGURE 5:**
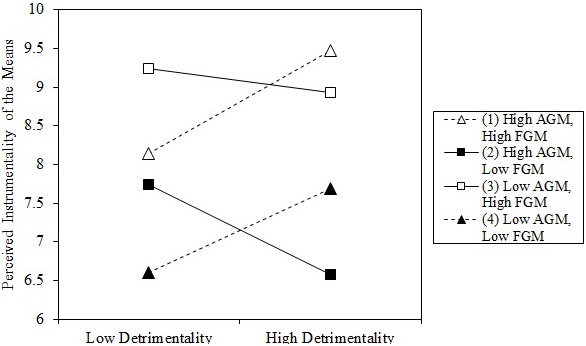
Instrumentality perceptions of getting tattooed (means) for low vs. high perceived detrimentality of the means dependent on alternative goal magnitude (AGM) and focal goal magnitude (FGM) (Study 5). High = one standard-deviation higher than the mean; Low = one standard-deviation lower than the mean.

To probe the nature of the three-way interaction, we computed the conditional effect of the Detrimentality × Alternative Goal Magnitude interaction for low vs. high levels of focal goal magnitude. The interaction Detrimentality × Alternative Goal Magnitude was significant for high levels (1 *SD* above the mean) of focal goal magnitude (*b* = 0.32, 95% CI [0.05,0.59], *t*(194) = 2.35, *p* = 0.020) as well as for low levels (1 *SD* below the mean) of focal goal magnitude (*b* = −0.52, 95% CI [−0.96,−0.08], *t*(194) = −2.35, *p* = 0.020). Akin to Study 4, we conducted simple slope analyses to probe the three-way interaction. Results indicated that the effect of detrimentality on perceived instrumentality was greatest for individuals high in alternative and focal goal magnitude (see **Figure 5**). Only the slope for high alternative and high focal goal magnitude (*b* = 0.55, 95% CI [0.87,1.01], *t*(194) = 2.34, *p* = 0.020) was significant (all other *p*s > 0.05).

Next, we tested the b-path in the model. Results indicated that the association between instrumentality and attitudes was significant, β = 0.27, *p* = 0.010. Lastly, bootstrapped confidence interval estimates of the indirect effect (see Preacher and Hayes, 2008) were calculated to confirm the significance of mediation. The 95% confidence interval of the indirect effect was obtained with 5000 bootstrap resamples (Preacher and Hayes, 2008). Results confirmed the mediating role of instrumentality between the predicting variables and attitude^3^ (β = 0.04; CI = 0.005–0.083).

In sum, Study 5 replicated the results of Study 4 by evincing the interactive role of detrimentality, alternative goal magnitude, as well as focal goal magnitude to predict perceived means instrumentality. Consistent with prior results, the more detrimental a means was to an alternative goal, the more it was perceived as instrumental to the focal goal. Importantly, these results were moderated by people’s commitment to the alternative and the focal goal. Moreover, we found that relatedness of the alternative and focal goal had no impact on the results, thus ruling out this alternative explanation in support of our model of counterfinality. Lastly, Study 5 demonstrated across various idiosyncratic goal-pursuits that higher perceived means instrumentality translates into greater liking for the means.

## General Discussion

Goals and their respective means of attainment are organized in associative networks (Kruglanski et al., 2002). Their activation potential follows a constant sum principle, whereby the greater the number of connections between these cognitive representations, the greater the dispersion of their activation (Anderson, 1983; Zhang et al., 2007). Prior work grounded in goal-systems theory has shown that adding a positive means-goal connection decreases the perceived instrumentality of the means (Zhang et al., 2007; Bélanger et al., 2015). We theorized that adding a negative means-goal connection would have the reverse effect. Specifically, we hypothesized that the more a means is detrimental to an alternative goal, the more it is perceived as instrumental to reaching one’s focal goal. This is the basic principle of counterfinality (Kruglanski et al., 2015). Additionally, we postulated that this phenomenon would be accentuated when highly valued alternative goals are neglected in the pursuit of important focal goals. Lastly, we posited that people would hold more favorable attitudes toward counterfinal means than non-counterfinal ones due to their increased perceived instrumentality.

In five studies, we found empirical support for the foregoing theoretical notions. In Study 1, people reported that getting tattooed was instrumental to their (idiosyncratic) focal goal as a function of the pain they experienced while being tattooed. This correlational study was corroborated in experiments that obtained additional support for our theoretical predictions. Specifically, in Study 2, participants presented with a counterfinal (vs. unifinal) mouthwash perceived it as more instrumental to the goal of fighting germs. The results indicated that this effect was mediated by the extent to which they believed using the mouthwash would create a burning sensation. In Study 3, we demonstrated that the counterfinality effect had psychological implications beyond that of shaping how means are perceived as effective for goal-pursuit. Replicating, as well as extending, Study 2, we found that counterfinal (vs. unifinal) products were perceived as more instrumental, and as a result, people held more favorable attitudes toward these products and reported greater intentions to purchase them. Studies 4 and 5 extended these findings by testing the boundary conditions of the counterfinality effect. In Study 4, we found evidence that the relationship between detrimentality and the perceived instrumentality of a means was moderated by alternative and focal goal magnitude. Specifically, the results indicated that the perceived instrumentality of a means peaked when the means was simultaneously instrumental to an important focal goal and detrimental to an important alternative goal. The latter finding highlights two important points: (1) neglecting important alternative goals enhances the costs associated with using a detrimental means and (2) without high focal goal magnitude, individuals are not motivated enough to neglect other important life domains and therefore do not perceive counterfinal means as the best course of action. Notably, the three-way interaction between detrimentality, alternative goal magnitude, and focal goal magnitude obtained in Study 4 was replicated in Study 5, in which participants generated an alternative goal to which the means was detrimental. This procedure had the methodological advantage of not forcing any goal-related content on participants, thereby demonstrating the breadth of our theoretical framework and its independence from specific goal content. Moreover, Study 5 replicated Study 3 by demonstrating that the perceived instrumentality of a means predicted positive attitudes toward the means, thus further supporting our model, for which theoretical and practical implications are considered below.

### Theoretical Implications

The present research extends prior work on goal-systems theory (Kruglanski et al., 2002, 2013b, 2015) and contributes to a burgeoning body of evidence that contends that the interconnections between means and goals have important self-regulatory implications (e.g., Köpetz et al., 2011, 2014; Orehek et al., 2012; Bélanger et al., 2016). Whereas ample research has compared the effects of unifinal and multifinal means, our study is one of the first empirical forays into counterfinality and thus provides support to the theoretical postulates of goal-systems theory and its nomenclature of means-goal configurations (Kruglanski et al., 2015).

Another contribution of this work is that the concept of counterfinality interweaves seemingly unrelated findings into a coherent framework. Indeed, different lines of research have shown that people gauge the effectiveness of means depending on whether the means appear to be illegal, effortful, painful, pricey, risky, unhealthy, unpleasant, and so on (e.g., Mazis, 1975; Labroo and Kim, 2009; Legare and Souza, 2012). However, all these are specific instances of a more general principle, namely that of counterfinality, under which they can be subsumed. Furthermore, contrary to previous research, our research indicates that the nature (or substance) of the alternative goal, to which the means is detrimental, is not relevant to observing increases in perceived instrumentality. Rather, what matters most is how goals and means are structurally connected. Study 5 highlighted the latter point by asking participants to elicit an idiosyncratic alternative goal to which tattooing was detrimental (e.g., comfort, job prospects, social approval, saving money). Despite the diversity of alternative goals, the more the means was detrimental, the more it was perceived as effective in advancing the focal goal.

One limitation of the current studies is that they offer only a momentarily picture of goal-means configurations and their consequences. However, motivational networks are malleable and dynamic. They fluctuate depending on situational influences; for instance, accessible information can influence which goal is most important and which means (e.g., product, activity) is perceived as the best course of action for goal pursuit. Thus, future research should experimentally manipulate goal magnitude. This could be done in the lab using goal priming and reaction time measures to further corroborate the cognitive mechanism behind the effect. Using goals that have no preexisting connections to a means (see Maimaran and Fishbach, 2014) could help understand whether the counterfinality effect occurs as an associative process independent of any learned heuristics. Lastly, future research should experimentally manipulate the order of the questions on detrimentality and instrumentality to test whether the counterfinality effect could be augmented by first making the negative aspects of an object or behavior more salient. For instance, this could be done replicating Study 1, while also including a condition in which people are first asked about the perceived effectiveness of getting tattooed and then about the amount of pain they experienced. Thereby, the sample size should also be increased to overcome limitations due to low power (which arguably might be the case for Studies 1 and 2).

Furthermore, the concept of counterfinality is of great relevance to the persuasion literature that compares the effectiveness of two-sided vs. one-sided messages on attitude change. In this regard, researchers (e.g., Pechmann, 1992; Bohner et al., 2003) have documented that messages that underscore both negative and positive attributes of a product (two-sided message) are more effective in creating positive attitudes toward the product than messages that expose only the positive attributes (one-sided message). However, the research on two-sided communication yielded mixed results, and several mechanisms have been proposed, such as the relatedness of positive and negative attributes (Pechmann, 1992; Bohner et al., 2003). The present research provides three novel contributions in relation to this literature.

First, the perceived instrumentality of a means, which increases following the disclosure of attributes detrimental to an alternative goal, can reliably explain *why* two-sided communication is more effective than one-sided communication in creating more positive attitudes. Second, results from Study 5 indicated that contrary to what has been suggested previously (e.g., Pechmann, 1992; Bohner et al., 2003), positive and negative attributes associated with a given means or product need not be correlated to an increase in the perceived instrumentality of the means. This is not to say that correlated attributes cannot reinforce each other to increase the perceived instrumentality of a means for a given goal (e.g., fat-content and food tastiness), but our results indicate that this is not a *necessary* condition for this phenomenon. Third, the magnitude of the effect of two-sided communication on attitudes is moderated by focal and alternative goal magnitude. This research is, to our knowledge, the first to consider the interaction between these factors and test the circumstances under which presenting negative information can succeed in increasing positive attitudes toward an activity or an object. Thus, the mixed results in the literature on two-sided communication might be due to the hitherto unconsidered varying importance of the focal and alternative goal. Taken together, the notion of counterfinality provides new insights into how negative information can have a positive impact on attitudes and ultimately influence the means people prefer for goal pursuit.

### Practical Implications

A long-standing question in consumer research is whether negative product information should be conveyed to consumers. Intuitively, one might consider downplaying or hiding negative product attributes. However, as the present research attests, negative information can be utilized to enhance the perceived effectiveness and hence the attractiveness of a given product or activity. One important related caveat is that for this approach to work, individuals must be highly committed to the focal and the alternative goals. For consumers less committed to the alternative or focal goals, it might be more effective to focus solely on positive attributes of the product.

Our findings are also relevant to health psychology. An interesting avenue for future research is whether placebo effects might be augmented by the inclusion of counterfinal information. For instance, research conducted by Espay et al. (2015) has documented improved motor function in patients with Parkinson’s disease when the patients used an expensive (vs. cheap) placebo. If placebo effects can be enhanced by the inclusion of counterfinal information (e.g., side effects, bitter taste), then the power of counterfinality could be harnessed to improve the life of others at virtually no cost.

The concept of counterfinality is also applicable to political psychology. Although voting behavior is a complex and multifaceted phenomenon (e.g., Todorov et al., 2005; Healy and Lenz, 2014), voters usually form their opinions based on how competent political candidates appear to be at solving important social, economic, and geopolitical issues. From this standpoint, voting for a political candidate or a specific ideology can be conceived of as a means to achieving a specific goal. If this is true, then it follows that voters would display a greater proclivity to vote for a political candidate who caters to their most important focal goal (e.g., fixing the economy) and especially so when the candidate is perceived as harmful to other important alternative goals (e.g., protecting religious minorities, saving the environment).

The same could be said for extreme forms of behaviors such as suicide terrorism or martyrdom (e.g., Kruglanski et al., 2013a, 2014; Bélanger et al., 2014), whereby individuals are willing to sacrifice their lives to further a political cause (e.g., ethnonationalist Tamil Tigers in Sri Lanka) or religious cause (e.g., jihadists of the Islamic State). Consistent with the findings herein described, it could be that militants and terrorists are (fatally) attracted to counterfinal means because these means are perceived as the most effective courses of action to reach their objectives. One question that begs future research is whether the appeal of counterfinal means can be minimized to help individuals leave terrorism behind. If so, what are the specific psychological interventions that could promote lasting change and reduce the risk of recidivism? These pressing questions await further investigation.

## Conclusion

The present research explored the phenomenon of counterfinality, whereby the more a means is detrimental to an important alternative goal, the more it is perceived as effective for goal attainment. Our findings indicated that counterfinality (1) was magnified for goal-pursuits involving important focal and alternative goals, (2) had significant implications for self-regulation by positively influencing people’s attitudes and behavioral intentions, and (3) accounted for a broad variety of psychological phenomena, including the perception that effortful means are effective, the appeal of forbidden activities or products, and the tendency of individuals to engage in self-harming behaviors. Overall, counterfinality is an integrative theoretical framework that provides a parsimonious explanation for the appeal of means with negative side effects.

## Ethics Statement

The data were collected in a manner consistent with ethical standards for the treatment of human subjects. Study 2 was conducted while the first author stayed at the University of Maryland (study was IRB approved). University of Maryland Institutional Review Board 315495-6 Preferences in daily life Reference number: 09-0317. The other studies were conducted while working at the Helmut Schmidt University in Hamburg, Germany (no IRB, ethics committee, or comparable institution available/required).

## Author Contributions

BS conducted the studies and wrote the manuscript. JB helped writing the manuscript and substantially contributed to the conception of the work. MD edited the manuscript and helped with analyzing and interpreting the data. H-PE revised the work for important intellectual content. AK contributed substantially with theory development and hypothesis generation.

## Conflict of Interest Statement

The authors declare that the research was conducted in the absence of any commercial or financial relationships that could be construed as a potential conflict of interest.

## Footnotes

1 The relatively small sample size (rendering the study slightly underpowered) is due to data collection being carried out close to the semester ending. Given that the first author had to leave the university she was visiting, it was not possible to collect more data.

2 The first author can be contacted to request the material.

3 Repeating the same analysis for a different attitude measure, i.e., “I love getting tattooed” yielded similar results.
